# Prevalence of *Plasmodium vivax *VK210 and VK247 subtype in Myanmar

**DOI:** 10.1186/1475-2875-9-195

**Published:** 2010-07-09

**Authors:** Tong-Soo Kim, Hyung-Hwan Kim, Sun-Sim Lee, Byoung-Kuk Na, Khin Lin, Shin-Hyeong Cho, Yoon-Joong Kang, Do-Kyung Kim, Youngjoo Sohn, Hyuck Kim, Hyeong-Woo Lee

**Affiliations:** 1 Department of Parasitology, College of Medicine, Inha University, Incheon 405-751, Republic of Korea; 2 Division of Malaria and Parasitic diseases, Korea Centers for Disease Control and Prevention, Seoul 122-701, Republic of Korea; 3 Vascular Medicine Research Unit, Brigham and Women's Hospital, Harvard Medical School, Cambridge, MA 02139, USA; 4 Department of Parasitology and Institute of Health Sciences, Gyeongsang National University College of Medicine, Jinju 660-751, Republic of Korea; 5 Department of Medical Research (Upper Myanmar), Pying Oo Lwin Township, Mandalay, Myanmar; 6 Faculty of Biomedical Engineering, Jungwon University, Goesan 367-805, Republic of Korea; 7 International Research Center of Bioscience and Biotechnology, Goesan 367-805, Republic of Korea; 8 Department of Gynecology, College of Oriental Medicine, Sangji University, Wonju 220-717, Republic of Korea; 9 Department of Pathology, University of Florida, J-566, 1600 SW Archer Road, Gainesville, FL 32610, USA

## Abstract

**Background:**

*Plasmodium vivax *is divided into two subtypes, a dominant form, VK210 and a variant form, VK247. This division is dependent on the amino acid composition of the circumsporozoite (CS) protein. In this study, the prevalence of the VK247 variant form of *P. vivax *was investigated in Myanmar.

**Methods:**

The existence of malaria parasites in blood samples was determined by microscopic examination, polymerase chain reaction (PCR) and DNA hybridization assays. To test for antibodies against *P. vivax *and *Plasmodium falciparum *in blood samples, an indirect immunofluorescence antibody test (IFAT) was performed using asexual blood antigens. An enzyme-linked immunosorbent assay with synthetic VK210 and VK247 antigens was carried out to discriminate between the *P. vivax *subtypes.

**Results:**

By thick smear examination, 73 (n = 100) patients were single infected with *P. vivax*, one with *P. falciparum *and 13 with both species. By thin smear, 53 patients were single infected with *P. vivax*, eight with only *P. falciparum *and 16 with both. Most of the collected blood samples were shown to be *P. vivax *positive (n = 95) by PCR. All cases that were positive for *P. falciparum *by PCR (n = 43) were also positive for *P. vivax*. However, 52 cases were single infected with *P. vivax*. IFAT showed antibody titres from 1:32 to 1:4,096. Additionally, using specific antibodies for VK210 and VK247, ELISA showed that 12 patients had antibodies for only the VK210 subtype, 4 patients had only VK247 subtype antibodies and 21 patients had antibodies for both subtypes. Using a DNA hybridization test, 47 patients were infected with the VK210 type, one patient was infected with VK247 and 23 patients were infected with both subtypes.

**Conclusions:**

The proportion of the VK247 subtype in Myanmar was 43.1% (n = 25) among 58 positive cases by serodiagnosis and 25.6% (n = 24) among 94 positive cases by genetic diagnosis. In both diagnostic methods, the infection status of malaria patients is highly diverse with respect to malaria species, and multiple clonal infections are prevalent in Myanmar. Therefore, the complexity of the infection should be considered carefully when diagnosing malaria in Myanmar.

## Background

*Plasmodium vivax*, a causative agent of relapsing benign tertian malaria, is the second most important malaria-causing species and afflicts several hundred million people annually [1]. Malaria constitutes a major health problem and is deeply associated with socioeconomic burden in many temperate and most tropical countries. In Myanmar, malaria is ranked as the number one public health problem, and nearly 600,000 malaria patients seek medical attention at health institutions annually. Among malaria species in Myanmar, *Plasmodium falciparum *accounts for 80% of infections, *P. vivax *accounts for 17.8% of infections and the remaining are due to *Plasmodium malariae *with mixed infections [2].

The surface membrane of all plasmodial sporozoites is covered by an antigen designated as circumsporozoite (CS) protein. The CS proteins have a central immunodominant region, consisting of tandemly repeated short amino acid sequences, which contain multiple copies of the immunodominant B cell epitope [3]. Because they are highly immunogenic and can induce a protective response in sporozoite-immunized experimental animals and in man, the CS proteins are being investigated as candidates for a human malaria vaccine. These immunodominant B cell epitopes within a large number of *P. falciparum *isolates of diverse geographical origin, and a smaller number of *P. vivax *isolates were examined and found to be conserved within the species [4]. A strain of *P. vivax *containing a variant repeat in its CS protein was first isolated in Thailand [5]. The CS repeat of this variant strain (Thai VK247) differs at 6/9 amino acids within the repeat sequence found in all previously described *P. vivax *CS protein. Following this discovery, several studies have been conducted to evaluate the global distribution of variant VK247; it was detected in indigenous populations of China [6], Brazil [7], Mexico [8,9], Peru [9,10], and Papua New Guinea [8].

It is known that the drug susceptibility of the VK247 subtype of *P. vivax *is slightly different than VK210 [11], as well as that *Anopheles albimanus *and *Anopheles pseudopunctipennis *differ in their susceptibilities to *P. vivax *circumsporozoite phenotypes. *Anopheles albimanus *is more susceptible to the VK210 subtype, whereas *An. pseudopunctipennis *is more susceptible to the VK247 subtype [12].

Here, the frequency of the two CSP variants (VK210 and VK247) in wild *P. vivax *isolates collected in Yangon and Mandalay, Myanmar was determined by ELISA and DNA hybridization. This effort may be useful for updating the anti-malarial control programme in Myanmar.

## Methods

### Study areas and blood collection of clinical isolates of malaria

Patients attending the Central VBDC (Vector Borne Diseases Control) clinic in Yangon and Wet-Won station hospital (Pyin Oo Lwin District) in Mandalay, Myanmar in January 2000 were selected for this study. All of the individuals exhibited clinical symptoms associated with malaria. Thin and thick blood smears were prepared from the blood collected for microscopic examination (magnification 7 × 100) from the fingertips of individuals. A local health unit employee read the Giemsa-stained thick blood smears in the fields, and treatment was administered to those individuals who tested positive for malaria, based on the guidelines of The Department of Health, the Union of Myanmar. And thin blood smears were examined by co-authors in laboratory condition. Before treatment, additional blood samples of approximately 3 ml were collected from each individual whose infection was confirmed by microscopic examination (n = 100). The blood samples were transferred to the National Institute of Health, Korea Centers for Disease Control and Prevention, Korea, for further polymerase chain reaction (PCR), DNA hybridization genotyping, and antibody analysis. Informed consent was obtained from all patients. The study protocol was approved by the Department of Health (Upper Myanmar), the Union of Myanmar.

### Amplification of CS the protein gene

For the purpose of preparing variant CS protein genes, genomic DNA was extracted from the whole blood of a malaria patient using the QIAamp Blood Kit (Qiagen Co., Hilden, Germany). PCRs were performed with the AccuPower PCR Premix (Bioneer Co., Taejeon, Korea), 50 ng of purified genomic DNA, 40 pmoles each forward (F1; 5'-TCCCCACGCACTGCGGGCACAAT-3') and reverse primers (R1; 5'-TTAATATGCACCGTGAGGACGCC-3'), and the total volume was adjusted to 20 μl with distilled water. The F1 primer contained several nucleotides of the signal peptide and the first 4 amino acids of the mature protein. The R1 primer contained the region starting 21 bases downstream of the stop codon so that this primer set would amplify the entire mature CS protein gene. The cycling conditions were performed as follows: denatured at 94°C for 5 min; 35 cycles of 1 min at 94°C, 1 min at 49°C, 2 min at 72°C; and a final incubation at 72°C for 5 min. A second PCR performed under the same conditions except that the PCR products (2-5 μl) were added instead of genomic DNA and that different forward (F2; 5'-AAAAAGGATGGAAAGAAAG-3') and reverse (R2; 5'-GACTTTTCATTTGGGGCA-3') primers were included in the reaction [13]. To exclude the blood samples that were co-infected with *P. falciparum*, PCRs were performed with a *P. falciparum *specific primer set that amplifies the ring-infected erythrocyte surface antigen (RESA), as described by Wooden et al [forward (5'-GATCAAGGAGGAGAGAACC-3') and reverse (5'-CAGCATTAACACCAACACC-3')] [14]. All the PCR products were analysed on a 1.2% agarose gel, confirmed under UV transillumination and purified with the NucleoSpin Extract kit (Macherey-Nagel, Duren, Germany). The purified PCR products were ligated with the pCR2.1 cloning vector (Invitrogen Co., Carlsbad, CA, USA) and then transformed into *E. coli *INVαF' according to the procedures of Invitrogen.

### Genotyping of PCR products by DNA hybridization

Half of the 20 μl PCR products was blotted onto a nylon membrane by a slot blotter. Two oligonucleotides, AL116 (5'-GGTGATAGAGCAGATGGA-3') for VK210 type and AL114 (5'-ATCAACCAGGAGCAAATG-3') for VK247 type, were used as probes in DNA hybridization [15]. These probes were end-labeled with ^35^S-α-dATP using a 3'-end labeling kit (Amersham Pharmacia Biotech, Uppsala, Sweden) for 1 hr at 37°C. The radiolabeled oligonucleotides were separated from unincorporated nucleotides on a Sephadex column (Amersham Pharmacia Biotech). Nylon membranes were soaked for 10 min in denaturation buffer and washed with distilled water, and then incubated with freshly prepared neutralization buffer two times for 15 min. After incubating the nylon membranes in 5× SSC (0.75 M sodium chloride and 0.075 M sodium citrate) for 5 min, the membranes were pre-hybridized for 1 hr at 42°C in 5× SSC, 1% SDS, 1× Denhart's solution (1% BSA, 1% Ficoll 400, and 1% polyvinyl pyrrolidone), and 5% sodium pyrophosphate. Hybridizations were performed in the same buffer with radiolabeled nucleotides for 12 hrs at 42°C. Each nylon membranes was co-reacted with anti-AL116 (5'-CCACTATCTCGTCTACCT-3') and anti-AL114 (5'-TAGTTGGTCCTCGTTTAC-3') for positive controls. After hybridization, nylon membranes were washed three times for 5 min each at 42°C in 5× SSC and 1% sodium pyrophosphate. These nylon membranes were air-dried and exposed to X-ray film (Kodak X-Omat, Sigma, St. Louis, MO, USA) with an intensification screen at -80°C for 72 hrs.

### DNA sequencing and analysis

After genotyping *P. vivax*, several candidates that could contain the variant CS protein gene were selected for DNA sequencing. After gel extraction with a Qiagen gel extraction kit, PCR products were ligated into the pCR2.1 cloning vector and transformed into *Escherichia coli *INVαF'. The PCR product inserted into *E. coli *was selected on ampicillin and 5-bromo-4-chloro-3-indolyl β-D-galactopyranoside (X-gal) containing medium. To determine whether the proper DNA was in *E. coli*, restriction enzyme digestion was done with *Eco*RI after plasmid isolation with a Qiagen plasmid isolation kit according to the method supplied by manufacture. The variant CS protein gene sequence was determined using the ABI PRISM dye terminator cycle sequencing ready reaction kit FS (Perkin Elmer, Cambridge, MA, USA) according to the supplied manual. M13 reverse and M13 forward (-20) primers were used in sequencing. Nucleotide and deduced amino acid sequences were analysed using EditSeq and Clustal in the Megalign program, and the multiple alignment programs in the DNASTAR package (DNASTAR, Madison, WI, USA). The internet-based BLAST search program of the National Center for Biotechnology Information (NCBI) was used to search protein databases.

### Indirect immunofluorescence antibody test

To test for antibodies against malaria, an indirect immunofluorescence antibody test (IFAT) was performed with whole blood antigens for *P. vivax *and *P. falciparum *as described by Sulzer *et al *[16]. Briefly, 10 ml of malaria parasite infected blood was collected by venopuncture from *P. vivax *and *P. falciparum *patients. After removing the plasma, the cells were suspended in phosphate buffered saline (PBS, pH 7.2) and centrifuged for 5 min at 2,500 rpm. The supernatant was discarded the cells were suspended in fresh PBS and the wash step was repeated three more times. Finally, an appropriate amount of PBS was added to maintain the parasitaemia at no less than 1%. The cells were dropped onto each well of Teflon coated slides. After drying them in room temperature for 12 hrs, the slides were kept at -70°C until required. To determine the antibody titres of each patient against *P. vivax *and *P. falciparum*, the antigen slides were fixed in pre-cooled acetone (-20°C) for 10 min, washed with PBS and added 20 μl diluted sera from 1:32 to 1:8,192 (vol/vol) was added to each well. The positive and negative controls were dropped on each slide and incubated in a moisture chamber for 30 min at 37°C. The reactions were stopped by washing out the reacted sera with PBS. The slides were immerged in PBS for 6 min and then dried at room temperature. Diluted FITC conjugated anti-human IgG (Sigma, 1:32 vol/vol in PBS) was added to each well and incubated and washed using the same method described above. Then several drops of buffered glycerol were added to the samples and covered with coverslips. The slides were examined under a 40× fluorescence objective.

### Enzyme-linked immunosorbent assay

To verify that the blood samples had antibodies against the synthetic VK210 and VK247 antigens obtained from R.A Wirtz (Centers for Disease Control and Prevention, Atlanta, GA, USA), an enzyme-linked immunosorbent assay was performed with these antigens. Briefly, 50 μl of capture antigen solution (1 μl antigen/50 μl) was placed in a 96-well plate (Corning, Lowell, MA, USA) and incubated for 12 hrs at room temperature. The cells were aspirated and filled with blocking buffer (1% BSA, 0.05% PBS-Tween 20) and incubated for 1 hr at room temperature. After washing the wells with 0.05% PBS-Tween 20 three times, the human serum samples in blocking buffer at a dilution of 1:100 (vol/vol) were added to each of the wells. The four positive and four negative control serum samples were also added to each plate. After a 2-hr incubation at room temperature, the plates were washed with 0.05% PBS-Tween 20 three times and then the peroxidase conjugated anti-human IgG (Sigma, 1:2,000, vol/vol) diluted in blocking buffer was added and incubated again for 1 hr at room temperature. The reaction was completed by washing the plates as described above. To develop the color, 100 μl of 2.2'-azino-di-(3-ethyl-benzthiozoline-6-sulfonic acid) (ABTS) peroxidase substrate (Kirkegaard & Perry Laboratories, Gaithersburg, MD, USA) was added and incubated for 30 min. The absorbance of the mixture was measured at 405 nm, and the cut-off value was taken as the mean ± 2 standard deviations of the negative samples.

## Results

### Characterization of each malaria parasite

To confirm malaria parasite infection from symptomatic patients, thin blood films were examined in fields and thin blood films were examined in the laboratory. As a results of the thick smear examination in the field, 73 patients (n = 100) were found infected with *P. vivax*, 14 with *P. falciparum*, and 13 with both species. By thin smear, 53 patients were infected with *P. vivax*, eight with *P. falciparum *and 16 with both species. Seven patients were shown to be negative by the thin smear technique.

Most of the collected blood samples were shown to be *P. vivax *positive (n = 95), and only five were negative by PCR examination. All patients who had *P. falciparum *also had *P. vivax *(n = 43). However, 52 patients were infected only with *P. vivax *(Table 1).

**Table 1:** Detection of malaria parasites by PCR with specific primers for *P. vivax *and *P. falciparum*.

Pv (Single)	Pf (Single)	Pv + Pf (Double)	Negative	Total
52	0	43	5	100

Pv; *Plasmodium vivax*

Pf; *Plasmoium falciparum*

In genotyping by DNA hybridization, 47 patients showed a positive reaction with VK210 (dominant form) specific probe, 23 patients were positive with both probes, and only one patient was positive for the VK247 subtype (variant form) (Table 2).

**Table 2:** Genotyping of *P. vivax *according to the nucleotide composition of their CS protein genes.

VK210^1^ (Single)	VK247^2^ (Single)	VK210+VK247^3^ (Double)	Not obtained^4^	Total
47	1	23	6	100

^1^; Patients who were infected with the *Plasmodium vivax *VK210 dominant form

^2^; Patients who were infected with the *Plasmodium vivax *VK247 variant form

^3^; Patients who were infected with the *Plasmodium vivax *VK210 and VK247 form

^4^; Sample could not be examined due to poor PCR reactions.

### Seroprevalence of malaria in Myanmar

When patient serum was analysed by IFAT that used *P. vivax *or *P. falciparum*-infected red blood cells as antigen, the titre against *P. vivax *ranged from 1:64 to 1:4,096. Ninety four patients showed high titre over 1:256 which represented current infection. Only six cases showed under 1:128. Seven samples did not have antibodies to *P. falciparum*, whereas the other samples had titres ranging from 1:32 to 1:4,096. Antibody titres of patients were distributed evenly rather than *P. vivax *cases and sixty seven cases showed over 1:256 (Table 3).

**Table 3:** Antibody titers of patients in response to *P. vivax *and *P. falciparum *asexual stage antigens.

	Reciprocal antibody titre									
		
	Negative	32	64	128	256	512	1,024	2,048	4,096	Total
Pv	0	0	1	5	11	18	36	24	5	100

Pf	7	2	13	11	10	14	12	18	13	100

Pv; *Plasmodium vivax*

Pf; *Plasmoium falciparum*

With regards to the proportion of antibodies against the variant form, 12 patients had antibodies for the VK210 subtype only, 21 patients had them for both and 4 patients only had VK247 subtype antibodies. Of the patients tested, 42 did not have any antibodies for either subtype, even they showed positive reaction in IFAT (Table 4).

**Table 4:** Serological typing of *P. vivax *isolates with circumsporozoite (CS) protein.

VK210^1^ (Single)	VK247^2^ (Single)	VK210+VK247^3^ (Double)	Negative	Total
12	4	21	42	100

^1^; isolates that reacted with synthetic peptide carrying the amino acid composition [GDRA(D/G)GQPA] of the dominant form of *P. vivax*.

^2^; isolates that reacted with synthetic peptide carrying the amino acid composition [ANGA(G/D)(N/D)QPG] of the variant form of *P. vivax*.

^3^; isolates that reacted with both types of *P. vivax*.

## Discussion

These studies demonstrated that parasite populations are highly heterogenetic and provided insight into the dynamics of disease transmission, which in turn drives our knowledge on drug application and disease control. Although Myanmar is one of the major malaria endemic countries and contributes to approximately 60% of malarial deaths in the South-East Asia region [2], the genetic diversity of the malarial parasites circulating in this county is poorly understood.

The *P. vivax *species contains two distinct polymorphisms, VK210 and VK247, which are widespread in Southeast Asia and South America [5]. In areas where two polymorphisms coexist, intrinsic biological differences between the polymorphs may affect their survival [17]. Fluctuations in the proportion of mosquitoes infected with the two polymorphs may reflect humoral immune pressure on the VK247 strain [18]. *Anopheles albimanus *was more susceptible to phenotype VK210 and less susceptible to phenotype VK247, whereas *Anopheles pseudopunctipennis *was the opposite. Additionally, the geographic distribution of the two polymorphs in southern Mexico reflects the distribution of the *Anopheline *mosquitoes, with patients living more than 170 m above sea level being infected with VK247 [19].

By microscopic examination, thick blood films can find more patients (n = 100) than thin blood films (n = 93), and these results might originate from the relatively small amount of blood used in the thin smear. Additionally, this examination missed seven cases of infection. However, we could determine malaria species more clearly in the thin smear. In the case of PCR examination, all patients were infected with *P. vivax*, except for five cases. Using this technique, any case was not infected only by *P. falciparum*: all were co-infected with *P. vivax *and P. falcipaum (Table 1). Additionally, positive rate in PCR was much higher than that of microscopic examination. Therefore, it is highly recommended that more intensive concentration is needed when examining the blood films in field and laboratory conditions. The fact that there were less *P. falciparum *cases than *P. vivax *cases is due to the season of blood sample collection, as *P. vivax *is predominant during the winter season in Myanmar [2].

During CS protein genotyping of *P. vivax*, one case was infected with only VK247, the variant form. Most of patients were infected with dominant form, VK210. Even though one case was infected with VK247 subtype only, a total of 23 cases (24.5%) were co-infected with VK247 and VK210 (Table 2). A similar distribution pattern of VK210 and VK247 mixed infections was observed in Thailand (25.6%) [20]. Although, all the cases of the variant form were not analysed, VK247 of Myanmar has high homology with the Thai strain (97.2%, GenBank accession No. U08983), it has a different number of nonapeptide repeats (i.e., Thai strain has 18 times repeat of ANGA(G/D)(N/D)QPG but the Myanmar strain has 19 repeats of AN(G/E)A(G/D)(N/D)QPG. This isolate has a different amino acid repeat in a third of the nonapeptide repeats, that is, usually the third amino acid is conserved as G, which is translated from GGA or GGG, however, in some of the repeats, it has an E instead of G, which is translated from GAG [5]. Interestingly, sporozoites carrying the VK247 sequence were more frequently produced in *An. albimanus *than sporozoites with the VK210 sequence [21]. Therefore, these results suggest that different vector species might be co-distributed in the study area and multiple infections by different *P. vivax *strains often occur in individuals throughout the study area, which is similar to the situation in Thailand [22].

Seroimmunological diagnosis, in particular by IFAT, is an important tool for the detection of malaria, especially when microscopic evidence of the parasites is not available due to the several reasons [23]. All patients had antibodies against whole blood antigens of *P. vivax *by IFAT. Among them, 94 cases (94%) had relatively high antibody titres above 1:256. In patients who were infected by *P. falciparum*, seven cases did not have antibodies against whole blood antigens. These cases might have been infected by *P. falciparum *recently. Additionally, the 67 cases had antibody titres above 1:256. These results indicate that most patients had been exposed to both species (Table 3). The number of antibody positive cases against VK247 (n = 25) antigen is similar to those determined by DNA hybridization (n = 24). In ELISA, Twenty-one cases showed mixed infection by VK210 and VK247, and 33 cases were positive for VK210 antigen. This finding indicates that VK247 transmission has actively progressed in Myanmar. There are 36 *Anopheline *mosquitoes in Myanmar [24], and there are differences in the sporozoite production of VK210 and VK247 in each vector mosquitoes [21]. Therefore, intensive field studies should be done in Myanmar to elucidate the relationship between *P. vivax *subtypes and sporozoites production rates in each *Anopheline *mosquitoe for malaria control in Myanmar.

## Conclusions

A major finding of this study was that the prevalence of malaria in Myanmar is complex like elsewhere; these data might be used as fundamental information for molecular epidemiological and seroepidemiological studies in Myanmar. The geographic location of Myanmar appears to contribute to the large diversity of *P. vivax *genotypes and serotypes in this country. Migration of people within the country and between the neighbouring countries may introduce a *P. vivax *population with different alleles, thereby resulting in the complexity of malaria parasites. Interallelic recombination between different parasite populations in the mosquito vector may also facilitate genetic variation of *P. vivax*. Further studies using a large number of blood samples collected from different geographic areas are required not only to determine the nationwide parasite heterogeneity and detailed malaria epidemiology in Myanmar but also to implement malarial control programmes in the country.

## Competing interests

The authors declare that they have no competing interests.

## Authors' contributions

TSK, HHK, and HWL conceived and designed the study and contributed to execution of research. TSK, HHK, and HWL wrote the manuscript. HWL, KL, BKN, YJK and DKK collected the blood samples in the field. YS, SSL, SHC and HK examined the thin blood films and KL thick blood film. YJK, DKK, YS and HK helped in the design and implementation of field studies. TSK, SSL, HHK and HWL carried out PCR, DNA hybridization, ELISA and IFAT. All authors have read and approved the final manuscript.
